# Whole-Genome Sequences of Two *Arthrobacter* sp. Strains, 4041 and 4042, Potentially Usable in Agriculture and Environmental Depollution

**DOI:** 10.1128/MRA.01054-18

**Published:** 2018-09-13

**Authors:** Julien Crovadore, Damien Grizard, Romain Chablais, Bastien Cochard, Philippe Blanc, François Lefort

**Affiliations:** a Plants and Pathogens Group, Research Institute, Land Nature and Environment, Hepia, HES-SO University of Applied Sciences and Arts Western Switzerland, Jussy, Geneva, Switzerland; b Proxis Développement, Levallois-Perret, France; c Bioprox, Noyant, France; University of Maryland School of Medicine

## Abstract

We report here the draft genome sequences of *Arthrobacter* sp. strains 4041 and 4042, both of which possibly belong to the diverse Arthrobacter agilis species and are potentially usable as plant biostimulants for agriculture and as depolluting bacteria for the environment.

## ANNOUNCEMENT

Arthrobacter spp. (Actinobacteria) are Gram-positive soil bacteria, appearing in a rod or coccoid shape (1), which grow under aerobic and anaerobic conditions (1). They are present in Antarctic (2) and desert (3) soils and in alkaline and subglacial lakes (4, 5), and some species are known to promote plant growth (5–8), to inhibit plant-pathogenic bacteria and fungi or wood-decaying fungi (7, 9), and to degrade a wide range of organic and polyaromatic pollutants (4, 10, 11).

These two strains were isolated from soil samples in western France. DNA was extracted with a modified cetyltrimethylammonium bromide (CTAB) protocol (12) from a pure culture grown exponentially from a single colony in LB broth. The sequencing library was built with the TruSeq Nano DNA PCR-free library preparation kit (Illumina, USA). Whole-genome sequencing was carried out within one Illumina MiniSeq run at a 2 × 151-bp paired-end read length using a MiniSeq high-output kit, with resulting genome coverages of 452× and 473× for strains 4041 and 4042, respectively. Overall quality metrics of the reads were assessed with FastQC version 0.11.5 (13). Genome assemblies were produced with SPAdes genome assembler version 3.10 (14), set in “paired-end assembly, careful mode,” and yielded 31 and 34 contigs (≥200 bp) for strains 4041 and 4042, respectively. They were finally ordered with BioEdit version 7.0.5 (15) and analyzed with QUAST version 4.6.3 (16) set as “QUAST: skip contigs shorter than 200 bp.” The total genome length was 3,878,126 bp with a GC content of 67.66% and an *N*_50_ value of 466,984 bp for the strain *Arthrobacter* sp. 4041 and 3,235,327 bp with a GC content of 68.85% and an *N*_50_ value of 391,935 bp for the strain *Arthrobacter* sp. 4042. A BLAST search of the complete 16S rRNA gene of these 2 strains showed that these strains share about 99.7% identity with several Arthrobacter agilis strains in the GenBank nucleotide database (17). Automated gene annotation was carried out by the NCBI Prokaryotic Genome Annotation Pipeline (PGAP) version 4.1 (18) and Rapid Annotations using Subsystems Technology (RAST) version 2.0 (19) using the ClassicRAST annotation scheme. PlasmidFinder version 1.3 (20) and plasmidSPAdes (21), both with default settings, did not detect any plasmids. PGAP identified 3,536 genes and 3,416 proteins in strain 4041 and 2,995 genes and 2,885 proteins in strain 4042. No known prophage was found. Based on the PGAP annotation, the NCBI genome neighbor report showed that strains 4041 and 4042 displayed 48.85% symmetric identity and 83.42% gapped identity with each other. Compared to the 2 other publicly available genomes, strain 4042 shared 99.85% symmetric identity and 99.99% gapped identity with *A. agilis* strain CGMCC 1.15723 from China, while 4041 displayed 56.82% symmetric identity and 86.29 gapped identity with strain UMCV2 from Mexico (8), confirming an observed high variability in the *Arthrobacter* genus (1). Their sequences also predicted resistance to antibiotics and toxic metal compounds. Strain 4041 has genes potentially involved in auxin synthesis and a nitrilase gene. Both strains are considered for agricultural and environmental uses.

### Data availability.

These whole-genome shotgun (WGS) projects were deposited at DDBJ/EMBL/GenBank under the accession numbers NFSC00000000 for *Arthrobacter* sp. strain 4041 and NFSD00000000 for *Arthrobacter* sp. strain 4042. The versions described in this paper are the first versions, NFSC01000000 and NFSD01000000. Concerning contigs, 31 and 34 contigs for *Arthrobacter* sp. strains 4041 and 4042, respectively, have been deposited at DDBJ/EMBL/GenBank under the accession numbers NFSC01000001 to NFSC01000031 and NFSD01000001 to NFSD01000034. Raw sequencing data sets have been registered in the NCBI Sequence Read Archive database (22) under the accession numbers SRR5513009 for strain 4041 and SRR5513012 for strain 2042.
